# Towards Transforming Neurorehabilitation: The Impact of Artificial Intelligence on Diagnosis and Treatment of Neurological Disorders

**DOI:** 10.3390/biomedicines12102415

**Published:** 2024-10-21

**Authors:** Andrea Calderone, Desiree Latella, Mirjam Bonanno, Angelo Quartarone, Sepehr Mojdehdehbaher, Antonio Celesti, Rocco Salvatore Calabrò

**Affiliations:** 1 IRCCS Centro Neurolesi Bonino-Pulejo, S.S. 113 Via Palermo, C.da Casazza, 98124 Messina, Italy; andrea.calderone95@gmail.com (A.C.); desiree.latella@irccsme.it (D.L.); mirjam.bonanno@irccsme.it (M.B.); angelo.quartarone@irccsme.it (A.Q.); 2 Department of Mathematics and Computer Sciences, Physical Sciences and Earth Sciences, University of Messina, 98124 Messina, Italy; sepehrbaher@gmail.com (S.M.); antonio.celesti@unime.it (A.C.)

**Keywords:** artificial intelligence, prognosis, diagnosis, neurorehabilitation

## Abstract

**Background and Objectives:** Neurological disorders like stroke, spinal cord injury (SCI), and Parkinson’s disease (PD) significantly affect global health, requiring accurate diagnosis and long-term neurorehabilitation. Artificial intelligence (AI), such as machine learning (ML), may enhance early diagnosis, personalize treatment, and optimize rehabilitation through predictive analytics, robotic systems, and brain-computer interfaces, improving outcomes for patients. This systematic review examines how AI and ML systems influence diagnosis and treatment in neurorehabilitation among neurological disorders. **Materials and Methods:** Studies were identified from an online search of PubMed, Web of Science, and Scopus databases with a search time range from 2014 to 2024. This review has been registered on Open OSF (n) EH9PT. **Results:** Recent advancements in AI and ML are revolutionizing motor rehabilitation and diagnosis for conditions like stroke, SCI, and PD, offering new opportunities for personalized care and improved outcomes. These technologies enhance clinical assessments, therapy personalization, and remote monitoring, providing more precise interventions and better long-term management. **Conclusions:** AI is revolutionizing neurorehabilitation, offering personalized, data-driven treatments that enhance recovery in neurological disorders. Future efforts should focus on large-scale validation, ethical considerations, and expanding access to advanced, home-based care.

## 1. Introduction

**Figure 1:**
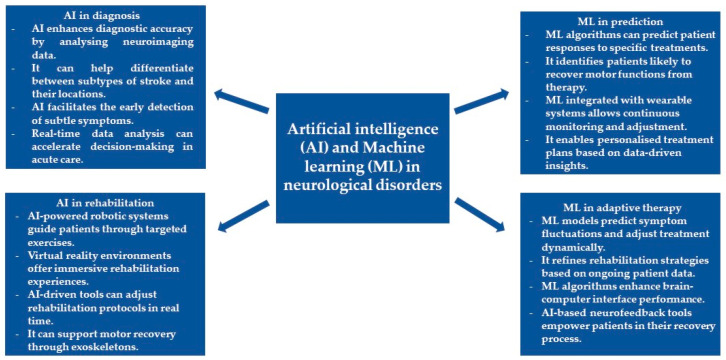
The benefits of AI in the diagnosis and neurorehabilitation of neurological disorders.

Neurological disorders represent a heterogeneous group of disorders in structure, biochemistry, and function within the nervous system, disrupting normal activity and giving rise to various symptoms and signs [1,2]. This is known to include traditional classifications such as neurodegenerative diseases, including Alzheimer’s and Parkinson’s disease (PD) [3,4], stroke [5], and traumatic injuries, such as spinal cord injury (SCI) [6]. Symptoms and signs often appear, but not always, with relation to the site of damage within the nervous system and may include mental deterioration, motor impairment, anesthesia, pain, and personality changes [7]. Most diseases are chronic and progressive; hence, the diagnosis, treatment, and patient care often result in overwhelming burdens [8]. Neurological disorders continue to stand as significant contributors to disability and mortality worldwide [9]. The World Health Organization estimates that almost a billion people have neurological disorders, thus placing a high burden on the disease [10]. Specifically, about 6 million people die annually due to stroke since it is among the major causes of death [11,12]. With high incidence, conditions such as stroke deserve care due to the acuteness and sometimes life-threatening nature of the conditions. Furthermore, stroke, SCI, and PD can affect both patients and caregivers. Therefore, proper management is needed to reduce disability and improve quality of life. Specifically, strokes and SCI require immediate medical attention and treatment to reduce damage to brain tissue [13,14,15,16], and proper rehabilitation may improve functional outcomes and quality of life. The progressive loss of neurons in PD leads to movement disorders, such as bradykinesia and tremors [17,18]. These issues experienced by patients become major challenges for doctors that should be addressed through good diagnosis and rehabilitation to slow the progression of the disease. For these neurological conditions, accurate diagnosis involving neuroimaging and neurophysiological tools is the most important step in guiding the mode of treatment and rehabilitation [19,20]. Neurorehabilitation itself subsequently becomes the major determinant in bringing about optimal recovery. Artificial Intelligence (AI) in the medical field has opened a new gateway to newer ways that concern the importance of diagnosis and timely neurorehabilitation in neurological disorders. These newer technologies can offer better rehabilitation outcomes and patient care through more personalized treatments based on data [21,22]. They play a critical role in the paradigm shift for neurorehabilitation due to the new avenues they avail for the understanding, diagnosis, and treatment of complex conditions such as stroke, SCI, and PD. AI, in general, is the performance of non-biological machinery on tasks that otherwise would have required cognition and, thus, intelligence from biological organisms [23]. Neurorehabilitation has increasingly used AI in its processes. By processing large volumes of data on patients, AI can detect patterns and predict rehabilitation outcomes that may not be evident to clinicians [24]. For example, AI-driven systems analyze either neuroimaging data or patient responses during rehabilitation and then make real-time adjustments to therapies to tailor interventions to meet the specific needs of individuals [25]. AI also provides the means for the creation of robotic exoskeletons and assistive devices that enable patients suffering from various forms of motor impairment to regain movement. AI could further enable virtual reality environments in which a patient can engage with immersive rehabilitative experiences that foster cognition and physical recovery [26]. This adaptability of AI is very important in rehabilitation situations, especially in conditions like stroke that depend on timely and precise intervention to minimize the continuity of a disabling condition [27]. It can be observed that where AI lays the broad overall framework, machine learning (ML) adds a more specialized layer to adaptability and prediction [28]. Focusing on ML indicates a preference for data-driven learning systems that can evolve constantly with the patient’s needs. ML is a part of AI and focuses on algorithms that, from themselves, learn from data through the identification of patterns in that given data to make predictions or decisions [29]. Therefore, neurorehabilitation takes priority in the provision of best outcomes through optimum recovery trajectory prediction using ML and finding the perfect rehabilitation strategies [30]. This means that ML models can, therefore, analyze such complex datasets of brain scans and motor function data to identify persons who are likely to respond to certain treatments [31]. For instance, ML algorithms can be used to predict which stroke patients are most likely to recover motor function from a specific therapy [32]. This is besides the fact that, in recent times, ML has increasingly been integrated into wearables and brain–computer interfaces, hence allowing for the real-time monitoring and modification of rehabilitation programs [33]. The latter functionality enables clinicians to tailor therapy as an integral part of a dynamic data-driven patient care process that changes with each session of therapy [34]. While understanding how these are being applied, it is essential to consider the combined power of AI and ML in real-world clinical settings. Especially in treating neurological disorders, the synergy between AI with broad analytical capabilities and ML with its predictive power is evident [35]. In the diagnostic field, AI helps to enhance the precision of diagnostic tools and develop more personalized treatment approaches, shaping a new era of care for people with these conditions [36]. These technologies enable early detection and quite accurate disease progression monitoring, with adaptive rehabilitation protocols that grow with the patient’s recovery for optimal short- and long-term outcomes [37]. AI is used in PD for early diagnosis and continual monitoring of motor symptoms with wearable devices, as well as to analyze data to predict symptom fluctuations and provide personalized treatment suggestions [38]. In stroke management, AI systems process neuroimaging data to rapidly identify stroke type and location, facilitating timely intervention to prevent further brain damage. AI can also help clinicians differentiate between ischemic and hemorrhagic strokes with high accuracy, aiding in selecting the most appropriate urgent treatment pathway [39,40]. In the field of rehabilitation, robotic systems, guided by AI, engage in physical therapy by guiding the stroke survivor through repetitive, task-oriented exercises promoting motor recovery through neuroplastic mechanisms [41]. Such systems immediately adjust to the patient’s performance to optimize movement patterns and enhance recovery of motor function [42]. AI-enhanced virtual reality environments allow for immersive simulations that cognitively and physically challenge patients in the restoration of complex motor and cognitive skills [43]. Additionally, ML methods are also being applied for functional recovery prediction and prognosis in post-stroke rehabilitation [44], further enhancing the personalized and adaptive nature of stroke recovery [45]. Applications of AI are fundamentally changing diagnosis and rehabilitation processes, even in cases of SCI. Concerning diagnosis, for example, AI algorithms can analyze MRI data to determine the extent of damage to the spinal cord and predict the potential recovery [46]. Further, AI can also provide an assessment of the function of nerves by incorporating electrophysiological data [47]. This kind of varied assessment enables a holistic understanding of the injury and, hence, can suggest treatment options [48]. In rehabilitation, AI-driven brain–computer interfaces and robotic exoskeletons allow the recovery of motor capabilities in SCI patients. These systems decode neural signals into movements, and correspondingly, these ML algorithms refine this process by learning from the patient’s interactions, thus increasing the accuracy and responsiveness of the technology over time [49,50]. Furthermore, AI-based neurofeedback tools allow patients to track their neural activity independently [51]. This instills active involvement in one’s recovery through brain response self-regulation for optimal outcomes in rehabilitation [52,53]. A summary of the benefits of AI and ML in the diagnosis and neurorehabilitation of neurological disorders is visualized in Figure 1.

This systematic review aims to investigate how AI tools are revolutionizing the diagnosis and treatment of neurological disorders, highlighting their transformative impact on neurorehabilitation strategies. The rationale behind this systematic review is that AI is increasingly affecting the medical domain, particularly in the diagnosis and treatment of neurological disorders. This is because of various AI tools, such as ML algorithms and neural networks, which guarantee rapid processing of voluminous data with high accuracy, thereby enabling more accurate diagnoses, predicting disease progression, and creating rehabilitation plans tailored to each patient’s needs. These technologies make neurological disorders increasingly detectable while revolutionizing neurorehabilitation with personalized treatments, optimized therapy schedules, and sometimes predictions of outcomes based on real-time data. Finally, AI has the capability for continuous learning from new data, thus enabling refinement in treatment approaches and offering unparalleled adaptability in patient care. A broad, supportive theoretical framework of this review is the concept of personalized medicine. When AI is adjunct to innovative technologies, including robotic devices, virtual reality, and metaverse, it can optimize motor recovery thanks to its adaptability to each patient. AI systems, through the analysis of vast amounts of patient data (e.g., medical history, injury type, progress metrics, and lifestyle factors), can facilitate the creation of customized rehabilitation programs that are tailored to each patient. 

Furthermore, AI is in line with the working principles of personalized medicine, where treatment is designed according to the unique biological and psychological attributes of each person along with environmental factors. That further justifies its relevance in the field. The current review tries to systematically address the role of AI in transforming diagnosis and therapy in neurological disorders, hence trying to provide a clear insight into how these innovations are revising neurorehabilitation strategies. Secondly, it attempts to outline the clinical utility of AI tools in the delivery of more effective, efficient, and patient-centered care. Therefore, in these times of increasing neurological disorders and limitations with the current methods, there is an emerging and urgent requirement to utilize all the features of AI in this crucial healthcare area.

## 2. Materials and Methods

### 2.1. Search Strategy 

**Figure 2:**
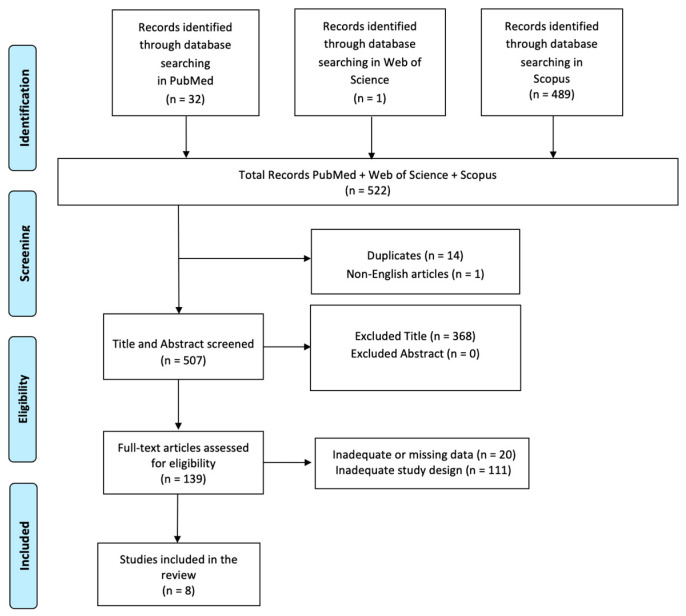
PRISMA 2020 flow diagram of evaluated studies.

A comprehensive literature search was performed using PubMed, Web of Science, and Scopus databases, employing the keywords: (All Fields: “Artificial Intelligence”) AND (All Fields: “AI diagnosis”) AND (All Fields: “AI neurorehabilitation”) with a search time range from 2014 to 2024. The PRISMA (Preferred Reporting Items for Systematic Reviews and Meta-Analyses) flow diagram was utilized to outline the process (identification, screening, eligibility, and inclusion) for selecting relevant studies, as illustrated in Figure 2. Titles and abstracts from the database searches were independently reviewed. Articles were evaluated for their relevance based on predefined inclusion criteria. All titles and abstracts that met these criteria were fully reviewed. Multiple expert teams independently selected articles and analyzed data to minimize bias, discussing discrepancies until consensus was achieved. This review has been registered on Open OSF (n) EH9PT.

### 2.2. PICO Evaluation

We applied the PICO model (Population, Intervention, Comparison, Outcome) to create our search terms. The population involved has a wide range of neurological conditions that are being treated with neurorehabilitation treatments. The intervention here is the incorporation of AI and ML in the diagnosis and treatment of the patient, showing how advanced systems are used to enhance clinical practice. The comparison is made with the traditional diagnostic and therapeutic approaches without AI or ML technologies. The primary outcome of such studies will be measuring the effectiveness of such AI and ML systems in improving diagnosis accuracy and treatment efficacy.

### 2.3. Inclusion Criteria

A study was included if it described or examined how AI and ML systems influence diagnosis and treatment in neurorehabilitation among neurological disorders. Only articles written in English were considered. Additionally, studies that described or investigated the functional assessment of these patients were included. We only included studies conducted in human populations and published in English that met the following criteria: (i) original or protocol studies of any kind and (ii) articles that examine how AI and ML systems influence diagnosis and treatment in neurorehabilitation among neurological disorders.

### 2.4. Exclusion Criteria

A study was excluded if it lacked data or information regarding how AI and ML systems influence diagnosis and treatment in neurorehabilitation among neurological disorders. Systematic, integrated, or narrative reviews were also excluded; however, their reference lists were reviewed and included when relevant. Additionally, any articles written in languages other than English were excluded. 

## 3. Results

A total of 522 articles were found: 14 articles were removed due to duplication after screening; 1 article was excluded because it was not published in English; 368 articles were excluded based on title and abstract screening. Finally, 131 articles were removed based on screening for inadequate and untraceable study designs (Figure 2). 

Therefore, eight research articles met the inclusion criteria and were included in the review. These studies are summarized in Table 1. 

The studies discussed in this review examine how AI and ML systems influence diagnosis and treatment in neurorehabilitation among neurological disorders. Eight articles analyzed the use of AI and ML in rehabilitating and diagnosing neurological disorders [54,55,56,57,58,59,60,61].

Murakami et al. conducted a study on how effective an AI-powered electromyography (EMG)-driven robot hand was in the rehabilitation process of the upper limbs in chronic stroke patients. The authors randomly assigned 20 participants to either an active or a control group; an active group underwent active finger training with the robot twice a week for four weeks. This significantly improved their motor performance and reduced the spasticity of the affected hemiplegic upper limb. The active group immediately showed and continued to demonstrate superior motor performance following intervention: the active group showed increased limb use and decreased wrist spasticity. Overall, the AI-driven robot hand effectively improved motor function and spasticity in chronic stroke patients for at least four weeks [54]. Another paper presented a novel computer vision system, which was developed to analyze the gait impairment of PD patients in a more sensitive and accessible way than traditional clinical measures. Deep learning extracted from videos captured by patients during regular assessments enabled detailed processing of motion data and estimation of gait severity after processing. Automatically created pre-assessments helped in error detection, allowing assessments without a physician. The results are in good agreement with severity assessments by expert physicians, and thus, it is a valuable tool for remote monitoring and assessment in PD patients [55]. Yang et al. created an AI model for diagnosing and tracking PD by analyzing night-time breathing patterns. Due to the lack of biomarkers for PD, the model was trained on a diverse dataset and performed efficiently in detecting PD with high accuracy. It estimated the severity and progression of PD accurately, simplifying the relationships between predictions and clinical assessments. The non-invasive measurement provided by the model enabled PD monitoring in a home environment, facilitating early risk assessments and clinical diagnosis. The interpretability of the AI model sheds light on how respiratory signals can reveal the symptoms of PD, making it a valuable tool for healthcare [56]. A retrospective study, however, aimed to improve the use of ML and explainable artificial intelligence (XAI) in predicting upper limb functional recovery after stroke rehabilitation. Random Forest algorithms and XAI methods were used to evaluate the prediction performance in subacute stroke patients. ML models outperformed classical statistical methods, providing better outcome predictions. XAI methods showed that baseline motor impairment quantified by clinical scales was critical to predicting recovery. Therefore, these results highlighted the dual benefit of ML and XAI, providing accurate predictions and transparent outputs for clinicians to make informed decisions on treatment strategies and monitor progress [57]. Mobbs et al. studied a novel ML model to assess arm movement abnormalities in patients with acquired brain injury during walking. They found that post-injury gait deficits were related to arm movement problems that affected psychological aspects. The International Classification of Functioning, Disability, and Health scale could assess these abnormalities but had moderate reliability. The researchers used gait videos to train ML networks to identify landmarks and joint angles to accurately predict clinical scores. The ML model performed similarly to human raters in predicting scores, with no significant differences between the different networks used. However, it slightly underestimated scores, suggesting further research on larger samples and objective assessments using smartphones or edge-based ML for better feasibility in local and remote rehabilitation [58]. A cross-sectional study examined the diagnostic potential of smartphones and smartwatches for detecting movement disorders, focusing on PD. Researchers collected data from 504 participants over three years, including PD patients, those with similar disorders, and healthy individuals. Participants used a smartphone app for interactive assessments, providing detailed movement data through questionnaires and smartwatch sensors. The study utilized ML to analyze the movement data, achieving high accuracy in distinguishing PD patients from healthy controls but lower accuracy in differentiating between PD and similar conditions. The dataset could be valuable for future ML research, potentially leading to a home-monitoring app for movement disorders and improving early diagnostics and treatment tracking [59]. Another paper used ML to predict gait recovery in SCI patients at discharge from a rehabilitation facility, which was the first in the field. More than a decade of patient data were analyzed using random forest and decision tree algorithms to predict walking ability, which proved to be accurate models. Initial walking ability, neurological classification, and somatosensory evoked potentials were key factors in recovery. These results may represent a decision support system to help clinicians predict gait recovery, enabling personalized rehabilitation strategies. ML shows potential to provide early prognosis and guide interventions tailored to individual patient needs, improving healthcare decision-making [60]. In a final study, ML techniques were utilized to classify stroke disabilities based on kinematic data from a Robotic Arm position-matching task. Proprioception impairment in stroke survivors was evaluated using a robotic system that measured 12 kinematic parameters. ML models were tested to see if they could classify stroke survivors more accurately than traditional clinical scoring methods. The study included stroke patients and healthy controls, with Random Forest and Deep Neural Networks algorithms used for classification. Results showed that ML models, particularly Random Forest, outperformed traditional scoring methods and achieved higher accuracy, sensitivity, and specificity in classifying stroke disabilities. Variability in movement was identified as a critical feature for accurate classification. This study highlights the potential of ML in processing complex kinematic data for better diagnosis and rehabilitation strategies for stroke survivors [61]. In essence, AI and ML have been strong tools in various emerging technologies for rehabilitation and diagnosis, improving diagnostic possibilities, personalization of therapies, and remote monitoring, thus opening routes for more precise interventions and optimized clinical management.

**Table 1:** Summary of studies included in the research.

Author	Aim	Study Design/Intervention	Treatment Period	Sample Size	Outcomes Measures	Main Findings
Murakami et al. 2023 [54]	To evaluate how using a robot hand integrated with AI and EMG technology affects upper extremity rehabilitation in chronic stroke patients.	Randomized Controlled Trial.	4 weeks.	20 patients.	FMA, MAL-14 AOU, MAS; H reflex and reciprocal inhibition.	The group that actively participated in the intervention demonstrated notable enhancements in FMA, MAL-14 AOU, and wrist MAS immediately after the intervention and also four weeks after. There were no notable enhancements observed in FMA for the control group.
Rupprechter et al. 2021 [55]	To assess a new computer vision technique using deep learning to measure the degree of walking problems in PD.	Methodological development study.	The study did not apply to treatment intervention, as it concentrated on creating and evaluating a gait assessment method.	The study utilized footage from 729 gait evaluations in which trained clinicians gave ratings.	The model’s ability to predict gait severity ratings were compared to clinician ratings, and the model’s predictions were also correlated with manual ratings.	The computer vision model achieved an accuracy of 50%, accurately estimating UPDRS ratings within one point of clinician ratings in 95% of cases. The model’s predictions showed a strong correlation with clinician diagnoses.
Yang et al. 2022 [56]	To create and assess an AI model to identify PD and monitor how it advances through analyzing night-time breathing patterns.	Development and evaluation study.	The study does not involve any treatment; rather, it focuses on the development and evaluation of AI models.	Data from 7671 individuals, encompassing information from various hospitals and multiple public datasets, was used to assess the model.	The AI model was evaluated based on its capacity to identify PD and to gauge the severity and advancement of PD.	The AI model can reliably identify PD and forecast its severity and progression. An attention layer is used for explainability and is capable of conducting remote PD assessments in homes without physical contact utilizing radio waves.
Gandolfi et al. 2023 [57]	To assess if ML can effectively forecast the recovery of UL function in patients recovering from sub-acute strokes and to pinpoint the key factors influencing these forecasts utilizing XAI techniques.	Retrospective study.	Patients received intensive, multidisciplinary upper limb rehabilitation for 2 h every day, 6 days a week, throughout their hospitalization. The mean period from stroke onset to release was around 37.71 days.	The ultimate dataset included 95 entries from a starting group of 192 individuals.	FMA-UE, TCT, MI, BI.	ML models outperformed standard statistical approaches in predicting UL recovery and the development of the illness. Baseline motor impairment was the most important characteristic. XAI techniques delivered reliable and clear findings, improving the comprehension of predictive variables.
Moobs et al. 2024 [58]	To determine the effectiveness of a novel two-tier ML model in detecting aberrant arm motions during walking in people with ABI.	Observational study.	Not specified.	42 ABI participants and 34 healthy controls.	Concordance between ML model predictions and clinician evaluations.	The ML model predictions were in close concordance with those of experienced human assessors, with no statistically significant variances between the networks. The models did not accurately forecast scores with minor impacts.
Varghese et al. 2024 [59]	To create reliable ML models for detecting and monitoring movement disorders using smart devices due to the lack of comprehensive datasets containing both movement data and clinical annotations for such disorders.	Cross-sectional study.	3 years.	504 participants, including individuals with PD, DD, and HC.	The outcome measure included the balanced accuracy of ML models in distinguishing between PD vs. HC and PD vs. DD, along with the detailed collection of clinical annotations and movement data.	The ML models obtained a mean balanced accuracy of 91.16% for distinguishing between PD and HC and 72.42% for distinguishing PD from DD. The research emphasizes the efficiency of the models but also acknowledges difficulties in differentiating between similar disorders.
Yoo et al. 2024 [60]	To forecast the restoration of walking ability post-SCI upon leaving a rehab center, utilizing ML methods to analyze crucial predictive factors and propose an ML-driven tool to aid in predicting gait recovery.	Retrospective Study.	Information was gathered between June 2008 and December 2021.	353 patients with traumatic or non-traumatic SCI.	The primary outcome was the FAC_DC.	The prediction of FAC_DC was accurate using random forest and decision tree algorithms, yielding RMSE values of 1.09 and 1.24 for all participants, 1.20 and 1.06 for traumatic SCI, and 1.12 and 1.03 for non-traumatic SCI. The primary factor for predicting gait recovery was found to be the initial FAC.
Hossain et al. 2023 [61]	To assess how stroke survivors perceive their body position using a robotic arm matching task and to evaluate the effectiveness of various ML methods and a task score in distinguishing between stroke survivors and non-stroke individuals based on movement data.	Cross-sectional study.	Not specified.	429 individuals who have had a stroke confirmed by neuroimaging (less than 35 days after the stroke) and 465 healthy individuals.	Parameters like trial-to-trial variability, spatial contraction/expansion ratio, systematic spatial shifts, and absolute error were used to measure performance in the arm position matching task. Task scores were additionally computed to evaluate overall effectiveness.	For the ML and deep learning models, the classification performance metrics were as follows: accuracy 82.4%, precision 85.6%, recall 76.5%, and F1 score 80.6%. Random Forest surpassed all other models in terms of numerical accuracy, scoring 83%. Both sensitivity and specificity were higher for ML models compared to the overall task score. Variability was the most dominant feature in classifying performance in the arm position matching task.

Legend: artificial intelligence (AI), electromyography (EMG), upper extremity (UE), Fugl–Meyer assessment (FMA), motor activity log-14 amount of use score (MAL-14 AOU), modified Ashworth scale (MAS), Parkinson’s disease (PD), Unified Parkinson’s Disease Rating Scale (UPDRS), machine learning (ML), upper limb (UL), explainable artificial intelligence (XAI), upper-extremity score on the Fugl–Meyer Assessment (FMA-UE), Trunk Control Test (TCT), Motricity Index (MI), Barthel Index (BI), acquired brain injury (ABI), differential diagnoses (DD), healthy controls (HC), spinal cord injury (SCI), decision support system (DSS), functional ambulation category at discharge (FAC_DC), root mean squared error (RMSE).

## 4. Discussion

This systematic review explored how AI, with regard to ML, influences diagnosis and treatment in neurorehabilitation. Indeed, evidence from the literature has increasingly emphasized the role that AI/ML could play in transforming motor rehabilitation and diagnosing neurological diseases. AI-based devices, including those with EMG-based robotic hands, have demonstrated significant improvements in upper limb motor function with a reduction of spasticity in stroke patients, reporting long-lasting results. Gait analysis in PD is improved by the application of ML models for greater accuracy, while functional recovery in stroke and SCI is driven forward more effectively with prediction. The analysis of complex kinematic data allows for a more precise classification of the degree of disability in stroke cases. Moreover, all these technologies improved diagnostic accuracy and personalized rehabilitation strategies and enabled remote monitoring [54,55,56,57,58,59,60,61]. Integrated into clinical care, AI offers the possibility of more frequent and specific interventions, effectively changing both the management and monitoring of neurological conditions from non-traditional healthcare settings. These results are confirmed by literature from different studies. Hashim et al. [62] introduced a stacking ensemble-based ML approach, further improving diagnostic accuracy by clustering multiple ML models. The improved performance of this approach over competing conditions of PD demonstrates that ensemble techniques can be applied in an advanced manner to the clinical setting [62]. Complementary to these insights, Wu et al. investigated wearable sensor devices that automatically detect the ON-OFF state of PD patients using interpretable ML models. This real-time capability supports dynamic adjustments in treatment and reflects the practical application of ML in PD monitoring and management [63]. Transitioning to stroke rehabilitation, Park et al. used clinical ML to find out who among stroke patients is the best responder to exoskeletal robotic gait rehabilitation. Their current study has shown that ML models can identify patients who will most likely benefit from this advanced therapy and enhance the personalization of rehabilitation protocols [64]. Carino-Escobar et al. went further to introduce strategies for session-to-session transfer learning in brain–computer interfaces during stroke rehabilitation. Their work highlights how adaptive learning techniques can further improve the performance of neurorehabilitation systems, staging the interventions according to the progress of individual patients [65]. Shifting focus to SCI, Håkansson et al. [66] studied data-driven approaches in predicting recovery outcomes for patients with SCI. Their study indicated the current capability and limitation of prediction models based on large datasets. More specifically, they underlined the capability of ML to improve the forecast of recovery by incorporating various clinical and physiological data [66]. Maki et al. proposed a web application based on ML algorithms for functional outcome prediction in traumatic SCI patients who are inpatients of rehabilitation centers. Their app is designed to process real-time patient data and provide personalized predictions about the success of rehabilitation. The study gives an example of how ML might work in practice to support clinical decision-making and personalized rehabilitation planning [67]. According to the studies presented in this review, we can also infer that AI and ML can achieve so much more than rehabilitation and diagnosis alone: they can predict recovery trajectories and inform clinical decisions [68,69]. These technologies can analyze a host of data that include patient history, demographic information, and the extent of neurological damage, offering predictive insight into how a patient might respond to specific treatments [70]. For instance, ML models predict the probability of a stroke patient achieving functional independence based on their performance during early rehabilitation [71,72]. Such outputs help clinicians set realistic goals and design appropriate therapy plans. In SCI, AI can predict if sensory or motor function will be regained in cases of injury [73,74,75]. This aids long-term insight into rehabilitation strategies. Furthermore, AI and ML are making neurorehabilitation even more accessible by becoming part of telerehabilitation platforms [76,77]. This advance enables constant monitoring and personalized treatment, even for patients residing in remote locations [78]. AI-powered systems may provide instant feedback during therapy sessions, that is, by changing exercises and guidance that do not require a therapist to be on-site [79]. Setting neurorehabilitation in this manner opens it towards more flexible, scalable, and patient-centered continuous support and adaptation concerning the patient’s progress [80]. Because of this, the field of neurorehabilitation and diagnosis of neurological disorders, including PD, stroke, and SCI, will experience a huge impact with the use of AI and ML. These technologies are improving not only the accuracy of early diagnosis but also the potential to create very personalized rehabilitation programs that adjust for the needs of every single patient [81,82,83,84,85]. With AI and ML, both diagnostic precision and treatment effectiveness stand to gain significantly [86,87,88,89]. In so doing, they are paving ways toward better patient outcomes, independence, and overall enhancement of the quality of life. As these technologies continue to evolve, their place within neurological care will likely grow and offer new hope for those afflicted by debilitating conditions [90,91]. 

Another important field of neurorehabilitation where AI and ML make their contribution is the treatment of non-motor symptoms. Non-motor symptoms from cognitive impairment and mood disorders to fatigue and chronic pain are often underestimated, though they seriously affect the quality of life in patients and complicate the process of rehabilitation. AI and ML can analyze big datasets emanating from electronic health records, wearable devices, and patient-reported outcomes to identify patterns that indicate the emergence or exacerbation of non-motor symptoms. For instance, predictive analytics can foresee impending cognitive decline among stroke survivors, thereby enabling clinicians to institute timely interventions that target improvements in cognition [92]. The early identification of cognitive impairment enables clinicians to allocate resources judiciously, targeting specific cognitive training exercises for those individuals while letting others pay attention to physical rehabilitation. Such an individual approach not only serves to further optimize treatment plans but also contributes to overall patient outcomes through the promotion of resilience. This kind of real-time monitoring by AI in PD could track the fluctuations in mood and cognition, and even medication adherence, for valuable insights to be gained by healthcare providers [93]. Advanced AI systems utilizing Natural Language Processing analyze conversations with patients in depth for subtle changes in the pattern of speech that may reflect mood disturbances [94]. This real-time feedback allows healthcare providers to make immediate adjustments to therapeutic interventions, improving both the effectiveness of the treatment and the overall patient experience. For example, clinicians can easily modify their therapeutic approach when anxiety or depression is observed in a patient by introducing supportive therapies or adjusting the medication regimen accordingly. Multiple sclerosis, among neurological disorders, highly predisposes patients to problems of fatigue, which also highly devastates day-to-day functioning and rehabilitation processes. AI-operated systems analyze the activity and behavioral patterns a patient demonstrates and make recommendations toward a carefully optimized schedule that balances active periods with rest as needed [95]. A patient may, through individual data, learn to cope better with energy-management habits for an improved quality of life. In addition, AI can provide cognitively impaired patients with natural language processing assistance so that communication with the patient can be facilitated during a clinical consultation. The patient is thus empowered to state his/her needs and concerns and can, therefore, adopt a collaborative approach to care [96,97,98].

Concerning the studies reported in this review, the results have a great many clinical implications. The findings of Murakami et al. [54] presented an AI-driven EMG-powered robot hand for chronic stroke patients and indicate that precise, targeted interventions may result in significant improvements in motor function and reduction of spasticity. This AI-driven intervention provides more individualization and adaptability of therapy, which is not so easily achievable with conventional rehabilitation therapy [99]. Importantly, sustained improvements in motor performance suggest that AI may make the therapeutic effects longer-lasting and may be linked to neuroplasticity within stroke rehabilitation [100]. The other critical finding pertains to gait analysis through deep learning and computer vision among patients with PD. Traditional clinical measures rarely have the sensitivity needed for such fine-grained assessment. The capability of AI in processing complex motion data opens new avenues for remote monitoring, enabling higher frequency and lower-cost assessments without any direct physician involvement, something that has been a gap in available care for patients with impaired mobility [101]. However, the full reliability of these systems compared to marker-based systems for motion analysis has yet to be determined.

The AI model by Yang et al., performing diagnosis of PD by analyzing night-time breathing, is another example of how AI can identify subtle, non-invasive biomarkers that current diagnostic tools may miss [102]. A very critical development that these studies have given prominent importance to involves the role of XAI and ML in predicting rehabilitation outcomes and recovery trajectories. The transparency in decision-making, whereby clinicians are not just using a “black box” algorithm but can understand and validate the rationale behind the predictions, is given by XAI in the prediction of upper limb functional recovery post-stroke [103]. This becomes an important factor in establishing trust in AI-driven healthcare tools. As the second example, ML applications for predicting gait recovery in SCI patients showed how AI could help in refining rehabilitation protocols by factoring in individual characteristics such as initial motor impairment and neurological classification, hence offering tailor-made, highly personalized care [104]. However, their clinical translation still needs further validation [105]. Although ML prediction models generally outperform traditional statistical methods, several studies have highlighted issues, including the underestimation of clinical scores and lower accuracy in distinguishing between PD and similar disorders. [106]. Larger datasets, more robust validation, and integration into everyday clinical practice are prerequisites for those technologies [107]. Similarly, while remote monitoring and home-based diagnostics are highly promising, there are still concerns regarding accuracy, patient compliance, and data security in that setting [108].

The management of neurological disorders also presents several issues regarding the ethics of informed consent concerning the use of AI and ML and the role of Institutional Review Boards (IRBs). Informed consent is a cardinal principle in medical studies and clinical practices wherein a patient is properly informed about participation in AI-driven studies or treatments. This means that patients should understand in what way their data will be used, what the possible risks and benefits are, and how AI technologies will be involved in their treatment. Only this level of transparency will contribute to gaining trust among the patients and service providers and may lead the patients to be more active in their treatment decisions [109]. Besides that, most AI technologies necessitate massive amounts of data, including sensitive personal health information. This further emphasizes the need for strict data protection. Not only is it essential to inform people about how their data will be collected, stored, and used, but also to address the risk of a data security breach. This helps to remove several privacy-related apprehensions and encourages more participation [110]. The IRBs, therefore, play a critical role in the oversight of research with AI and ML, giving due emphasis to patient rights and welfare. They ensure that studies are ethically conducted, data privacy and security are assessed, and the potential for algorithmic bias is reviewed [111]. Algorithmic bias in AI can lead to disparities in care that will significantly affect vulnerable populations of neurological disorders, making such IRB oversight quintessential in identifying issues and measures that may be required. IRBs are also charged with the duty of ensuring that processes of informed consent are meaningful and accessible in cases when research participants suffer from impairments in cognition or barriers to communication, something quite common in neurological disorders [112]. This should include the use of simple language and support to explain some of the complex issues regarding AI and ML. Through rigorous study designs and AI methodologies, IRBs also help minimize risks associated with untested algorithms and improve patient safety. They also stand at the forefront in demanding ethical standards matching this unparalleled technological advance.

This systematic review has important strengths: a thorough search across multiple databases to capture as many relevant studies as possible and utilizing the PRISMA framework to improve transparency and rigor; the PICO model underpins structuring the review around specific clinical questions related to AI and ML in neurorehabilitation. The focus on a range of neurological disorders, such as stroke, SCI, and PD, adds clinical relevance to the review. Emphasizing real-world applications in AI and ML, like robotic systems and brain–computer interfaces, serves to bring forth the transformational potential of these technologies in making treatments personalized and rehabilitation outcomes better. 

The limitations of this review are that because there were only eight included studies, the findings might not be generalized easily. Additionally, the majority of studies are temporary, so the effectiveness over a long period is unknown. While the exclusion of preclinical studies may be an effort to ensure clinical relevance, it may not be considered important foundational work. Heterogeneity in both methodologies and patient populations complicates the drawing of uniform conclusions. Lastly, the limited number of standardized outcome measures, combined with the absence of any cost-effectiveness analysis, limits wider applicability.

## 5. Conclusions

In conclusion, AI and ML, in particular, are greatly changing the outlook on diagnosis and rehabilitation in neurological disorders, especially in stroke, SCI, and PD. These can offer earlier and more accurate diagnoses, allowing personalized treatment strategies that might considerably improve outcomes for the patients. AI processes big volumes of data, while ML foretells the outcomes and provides valuable insights for clinicians to tailor rehabilitation. Robotic systems, interfaces of brain–computer, and virtual reality build a commonly integrated environment that enhances neuroplasticity and promotes effective rehabilitation with precision. In addition, AI-driven and ML-based telerehabilitation extends the outreach of services at real-time adaptive interventions, even at very remote distances. The future direction entails more studies that could help ensure the full-scale integration of AI and ML into routine clinical practice. Clinical trials on a large scale should be carried out to validate the efficacy and safety of AI-driven rehabilitation tools in diverse patient populations. Developmental efforts must focus on the creation of scalable and user-friendly systems that could easily fit into health settings. Among the ethical issues to be considered are data privacy, algorithm transparency, and the possibility of bias in the AI models. Taking these measures will ensure that all patients receive fair and equal care. To date, the integration of AI and ML in home-based rehabilitation systems may democratize access to advanced care by offering real-time feedback and personalized interventions to those patients with limited access to specialized rehabilitation centers. In this view, the future of neurorehabilitation will be molded by an ever-enhancing capability of AI and ML to improve neurological recovery and improve long-term outcomes in patients.

## Data Availability

The data that support the findings of this study are not openly available due to sensitivity reasons and are available from the corresponding author upon reasonable request.
